# Theoretical Study and Analysis of CsSnX_3_ (X = Cl, Br, I) All-Inorganic Perovskite Solar Cells with Different X-Site Elements

**DOI:** 10.3390/molecules29112599

**Published:** 2024-05-31

**Authors:** Shiyu Yuan, Zhenzhen Li, Yitong Wang, Hang Zhao

**Affiliations:** College of Metallurgy and Energy, North China University of Science and Technology, 21 Bohai Street, Tangshan 063210, China; yuanshiyu9999@163.com (S.Y.); wangyitongjilin@163.com (Y.W.)

**Keywords:** CsSnX_3_ (X = Cl, Br, I), all-inorganic perovskite, solar cell, SCAPS-1D simulation software, performance optimization

## Abstract

In this research, SCAPS-1D simulation software (Version: 3.3.10) was employed to enhance the efficiency of CsSnX_3_ (X = Cl, Br, I) all-inorganic perovskite solar cells. By fine-tuning essential parameters like the work function of the conductive glass, the back contact point, defect density, and the thickness of the light absorption layer, we effectively simulated the optimal performance of CsSnX_3_ (X = Cl, Br, I) all-inorganic perovskite solar cells under identical conditions. The effects of different X-site elements on the overall performance of the device were also explored. The theoretical photoelectric conversion efficiency of the device gradually increases with the successive substitution of halogen elements (Cl, Br, I), reaching 6.09%, 17.02%, and 26.74%, respectively. This trend is primarily attributed to the increasing size of the halogen atoms, which leads to better light absorption and charge transport properties, with iodine (I) yielding the highest theoretical conversion efficiency. These findings suggest that optimizing the halogen element in CsSnX_3_ can significantly enhance device performance, providing valuable theoretical guidance for the development of high-efficiency all-inorganic perovskite solar cells.

## 1. Introduction

With the continuous advancement of technology and the reduction in costs, solar cells are becoming increasingly popular as a feasible energy solution, especially in the current global energy transition [1]. Among them, all-inorganic perovskite solar cells are a research hotspot in the field of solar cells. This is because all-inorganic perovskite solar cells have significant advantages in terms of photoelectric conversion efficiency (PCE), stability, and cost-effectiveness [2,3]. Unlike traditional organic–inorganic hybrid perovskite materials, all-inorganic perovskites exhibit enhanced thermal and photo stability. It is worth noting that CsSnX_3_ (X = Cl, Br, I) perovskite has attracted widespread attention due to its small lattice distortion, suitable band energy levels, and non-toxic properties of tin ions [4].

The CsSnX_3_ (X = Cl, Br, I) all-inorganic perovskite material has a typical ABX_3_ cubic crystal structure, in which the Cs^+^ ion is located at the A-site, the Sn^2+^ ion is located at the B-site, and the Cl^−^, Br^−^, and I^−^ ions are located at the X-site [5]. The stability of this structure makes all-inorganic perovskite materials exhibit good photoelectric properties in photoelectric conversion devices. The excellent photoelectric properties of CsSnX_3_ all-inorganic perovskite materials are mainly due to their special crystal structure and electronic energy level layout. The photoelectric properties of CsSnX_3_ perovskites can be effectively regulated by adjusting the composition of X elements (Cl, Br, I). As the X element shifts from Cl^−^ to I^−^, the band gap energy levels of CsSnX_3_ perovskites progressively decrease, and the absorption edges from the ultraviolet to visible regions also move to the long wavelength direction accordingly, so the device’s performance is improved to varying degrees [6].

Recent advancements in the study of CsSnX_3_ (X = Cl, Br, I) all-inorganic perovskite devices have shown notable progress. Scientists have enhanced the crystal quality and photoelectric performance of these devices through optimized preparation methods and interface engineering, resulting in improved photoelectric conversion efficiency (PCE). For instance, refining the preparation process can effectively enhance the crystalline quality and photoelectric characteristics of CsSnX_3_ all-inorganic perovskite films, promoting the development of all-inorganic perovskite devices. In addition, doping appropriate ions or molecules, such as Pb, Bi, N, etc., can also regulate the energy level structure and carrier transport properties of CsSnX_3_ perovskites to further improve the performance of the device [7]. Xu’s group optimized the charge extraction efficiency by adding intermediate energy levels, thus obtaining a CsSnBr_3_ all-inorganic perovskite device with a PCE of 9.13%, and it showed ultra-high stability at 80 °C for more than 720 h [8]. In 2024, Wang’s group used a bismuth ion (Bi^3+^)-doped B-site to optimize CsSnI_3_ perovskite to improve its performance. The incorporation of Bi^3+^ into Sn^2+^ can enhance the morphology, crystallinity, and stability of perovskite films while decreasing trap density. The CsSnI_3_ all-inorganic perovskite devices with a PCE of 6.11% were successfully prepared in this way, and the initial efficiency of 72% remained unchanged even after 240 h of storage [9]. In addition, Chen’s group prepared CsSnI_3_ quantum rods by a solvothermal process, and they obtained all-inorganic perovskite devices with a PCE of 12.96% [10]. However, although CsSnX_3_ (X = Cl, Br, I) all-inorganic perovskite devices have much research progress, the energy band matching, work function, defect secret, and thickness of CsSnX_3_ all-inorganic perovskite materials and hole and electron materials have not been optimized, which also leads to lower device efficiency than organic–inorganic hybrid perovskite materials.

Hence, to enhance the performance of CsSnX_3_ (X = Cl, Br, I) all-inorganic perovskite devices featuring various X-site halogen elements, SCAPS-1D simulation software (Version: 3.3.10) was employed for the theoretical prediction of device structure and parameters. According to the simulation results, by changing the hole and electron materials, the energy band matching degree with the CsSnX_3_ perovskite layer can be effectively changed, and the device efficiency can be improved. Optimizing parameters such as work function, defect density, and thickness of the optical absorption layer can lead to further enhancement in device performance. In addition, this study further proves that the device performance will be greatly improved with the sequential substitution of halogen elements (Cl, Br, I). These theoretical results provide interesting theoretical guidance for the experimental study of CsSnX_3_ all-inorganic perovskite devices.

## 2. Results

### 2.1. Device Model Verification

In this article, the physical parameters of each layer, as utilized in SCAPS-1D simulation software, are sourced from previously published experimental and basic theoretical research, as demonstrated in Table 1. And we set the electron and hole cross sections captured at the interfaces of HTL/CsSnX_3_ and CsSnX_3_/ETL to 1 × 10^−19^ cm^2^ (other parameters are set as shown in Table 2). The thermal velocity and captured electron and hole cross sections of each layer of the device are set to 1 × 10^7^ cm/s and 1 × 10^−15^ cm^2^, respectively. In addition, for the light absorption coefficient of each layer, the light absorption formula α = A_α_(hv − E_g_)^1/2^ provided in the software is used for calculation, and the absorption constant A_α_ is maintained at its default value of 1 × 10^5^. To enhance the reliability of the simulation outcomes, the work function of the CsSnX_3_ all-inorganic perovskite device is set to 5.1 eV (Au) and 4.75 eV (ITO), respectively. Meanwhile, the simulation conditions are conducted under standard conditions (under the AM1.5 spectrum with a light intensity of 1000 W·m^−2^).

**Figure 1:**
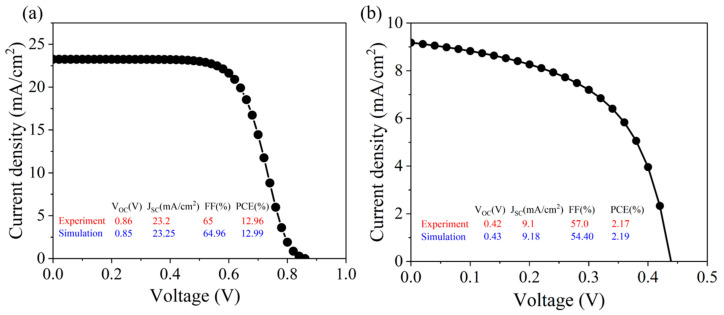
The volt-ampere (J-V) characteristic curve of all-inorganic perovskite solar cells: (**a**) CsSnI_3_; (**b**) CsSnBr_3_; experimental values from the literature [10,11].

This study uses the physical parameters in Table 1 to verify the reliability of the simulation tool. Firstly, CsSnI_3_ with a small band gap is selected as the light absorption layer for verification. The characteristic parameters of the CsSnI_3_ all-inorganic perovskite device are acquired through simulation, as depicted in Figure 1a. The results are very similar to the experimental results and device structure reported by Chen’s group in 2016 [10]. However, in order to avoid the contingency in the experiment, the light absorption layer material was replaced by CsSnBr_3_ in this study. The conductive glass ITO (4.75 eV) of the all-inorganic perovskite device was replaced by FTO (4.4 eV), and the defect density at the interface was designated as 1 × 10^16^ cm^−3^. The simulation outcomes closely align with the experimental findings of the FTO/TiO_2_/CsSnBr_3_/Spiro-OMeTAD/Au all-inorganic perovskite device employed by Gupta’s group in 2016 [11], as shown in Figure 1b. This serves to reinforce the credibility of the SCAPS-1D simulation software utilized in this study. Consequently, this research will utilize the SCAPS-1D software for the numerical simulation of CsSnX_3_ (X = Cl, Br, I) all-inorganic perovskite devices.

**Table 1:** Physical parameters of TiO_2_, CsSnX_3_ (X = Cl, Br, I), and Spiro-MeOTAD.

Parameters	TiO_2_	CsSnCl_3_	CsSnBr_3_	CsSnI_3_	Spiro-MeOTAD
Thickness (nm)	25	240 *	150 *	177 *	100
Energy band E_g_/(eV)	3.2	2.8	1.75	1.3	2.8
Electron affinity χ/(eV)	4.0	3.9	4.07	3.6	2.05
Relative permittivity ε_r_	9.0	29.4	5.9	9.93	3
Effective conduction band density N_c_/(cm^−3^)	1.0 × 10^21^	1 × 10^19^	1 × 10^18^	1 × 10^18^	2.2 × 10^18^
Effective valence band density N_v_/(cm^−3^)	2.0 × 10^20^	1 × 10^19^	1 × 10^18^	1 × 10^19^	1.8 × 10^19^
Electron mobility μ_n_/(cm^2^·V^−1^·s^−1^)	20	2	0.1	1500	0.0002
Hole mobility μ_p_/(cm^2^·V^−1^·s^−1^)	10	2	0.1	585	0.0002
Donor density N_D_/(cm^−3^)	1 × 10^18^	0	0	0	0
Receptor density N_A_/(cm^−3^)	0	1.0 × 10^15^	1.0 × 10^15^	1.0 × 10^18^	1.0 × 10^18^
Defect density N_t_/(cm^−3^)	1 × 10^15^	1.0 × 10^15^ *	1.0 × 10^16^ *	1.0 × 10^15^ *	1.0 × 10^15^
Reference	[12]	[13]	[14]	[15]	[15]

All the data with * in the table are simulated initial variables.

**Table 2:** Defect parameters in the interface.

Parameters	HTL/ CsSnCl_3_	CsSnCl_3_/ ETL	HTL/ CsSnBr_3_	CsSnBr_3_/ ETL	HTL/ CsSnI_3_	CsSnI_3_/ ETL
Defect type	Neutral	Neutral	Neutral	Neutral	Neutral	Neutral
Electron capture cross section (cm^2^)	1 × 10^−19^	1 × 10^−19^	1 × 10^−19^	1 × 10^−19^	1 × 10^−19^	1 × 10^−19^
Hole capture cross section (cm^2^)	1 × 10^−19^	1 × 10^−19^	1 × 10^−19^	1 × 10^−19^	1 × 10^−19^	1 × 10^−19^
Energy distribution	Gaussian	Gaussian	Gaussian	Gaussian	Gaussian	Gaussian
Reference defect	0.6	0.6	0.6	0.6	0.6	0.6
Characteristic energy	0.1	0.1	0.1	0.1	0.1	0.1
Total defect density (cm^−3^)	1 × 10^11^	1 × 10^11^	1 × 10^16^	1 × 10^16^	5.5 × 10^9^	5.5 × 10^9^

### 2.2. The Effect of Conductive Glass and the Back Contact Point Work Function

The work function (φ) stands as a crucial factor impacting the efficacy of CsSnX_3_ (X = Cl, Br, I) all-inorganic perovskite devices. A suitable difference in work function can facilitate effective electron transmission and collection, thereby enhancing solar cell efficiency. A lower disparity in work function can diminish interface impedance, foster electron injection and transmission, and improve electron carrier mobility. Additionally, the work function also influences solar cell performance. The difference in work function between the contact point and the all-inorganic perovskite layer can affect carrier injection and transport efficiency. A suitable difference in work function can enhance charge collection and reduce charge recombination and loss, thereby boosting solar cell efficiency. Hence, in the design of CsSnX_3_ all-inorganic perovskite devices, it is imperative to select appropriate conductive glass and back contact points to achieve a work function difference that aligns with the band structure of CsSnX_3_ (X = Cl, Br, I) materials. This optimization can maximize charge transfer and collection efficiency, ultimately improving solar cell performance and efficiency. Therefore, this chapter delves into the influence of these two parameters on the performance of all-inorganic perovskite devices.

#### 2.2.1. Conducting Glass

Conductive glass FTO (tin fluoride-doped tin oxide) and ITO (tin-doped indium oxide) are two commonly used conductive glass materials, which are widely used in the field of photovoltaic devices [16]. The work function ranges of FTO and ITO are generally about 4.4 eV and 4.8 eV, respectively, and they have good transparency and conductivity. Therefore, in the design and preparation of solar cells, the selection of appropriate conductive glass materials can effectively improve the device’s performance [17].

Since the conductive glass FTO may be more stable than ITO in some specific environments, especially in high-temperature or acidic environments, FTO has higher durability. In addition, the price of FTO is usually cheaper than ITO, which makes FTO more attractive in some commercial applications, especially in cost-sensitive large-scale production. Therefore, based on FTO conductive glass, this study simulates the effects of different work functions on the performance of a CsSnX_3_ (X = Cl, Br, I) all-inorganic perovskite device. The device structure used in this study is FTO/TiO_2_/CsSnX_3_/Spiro-OMeTAD/Au.

**Figure 2:**
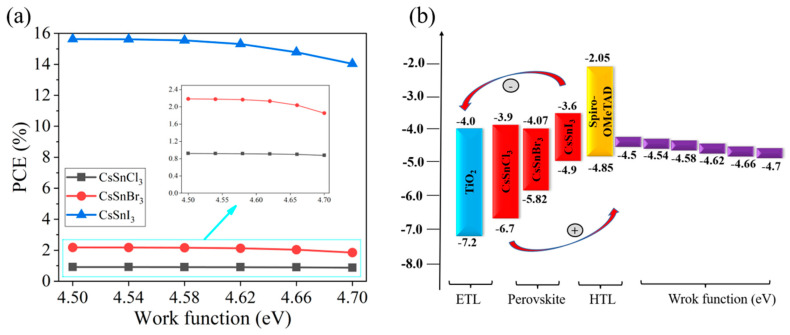
Different work functions: (**a**) photoelectric conversion efficiency; (**b**) energy band diagram.

This study simulates the performance changes of different devices when the work function of the conductive glass FTO increases from 4.5 eV to 4.7 eV, as shown in Figure 2a,b. The simulation results indicate a notable decrease in the PCE of the CsSnX_3_ all-inorganic perovskite device with the elevated FTO work function. This decline can be ascribed to the amplified energy band offset between the conductive glass and the all-inorganic perovskite film as the FTO work function increases, leading to heightened barriers and reduced efficiency in electron injection and collection. This results in an escalation of interface resistance, impacting the current transmission efficiency of the device. Consequently, the device performance declines as the FTO work function increases. Therefore, an appropriate work function of conductive glass can effectively improve the device’s performance. When the FTO work function is less than or equal to 4.54 eV, CsSnX_3_ (X = Cl, Br, I) all-inorganic perovskite devices have high PCE. Thus, in this study, the FTO work function is fixed at 4.54 eV, and optimization efforts proceed from this established parameter.

#### 2.2.2. Back Contact Point

**Figure 3:**
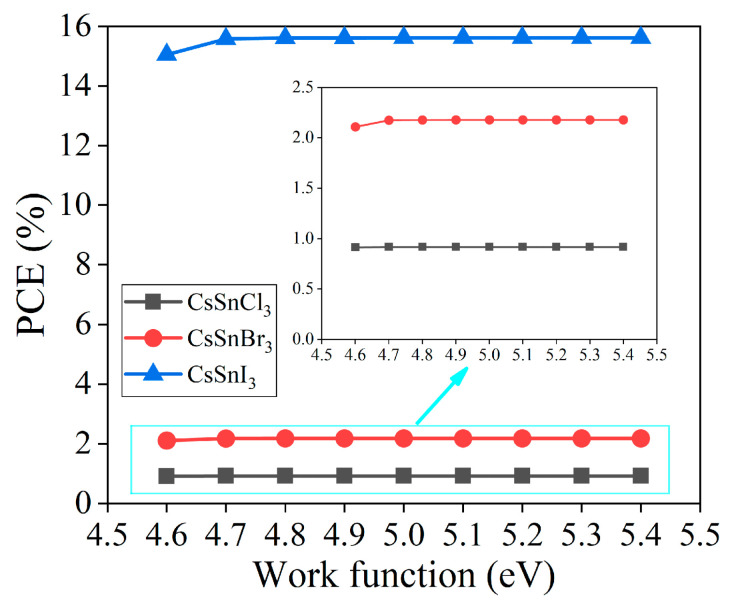
Photoelectric conversion efficiency of different back contact point work functions.

The difference in the work function between the back electrode and the energy level in the perovskite structure profoundly affects the performance and efficiency of CsSnX_3_ all-inorganic perovskite devices [18]. This study incrementally increases the work function of the back electrode from 4.6 eV to 5.4 eV using the simulation software SCAPS-1D to investigate the performance variations of CsSnX_3_ all-inorganic perovskite devices. Figure 3 is presented to analyze the trend of photoelectric conversion efficiency in CsSnX_3_ (X = Cl, Br, I) solar cells with changes in the work function.

Based on the simulation outcomes, enhancing the performance of the CsSnX_3_ all-inorganic perovskite devices is observed when the work function of the back electrode exceeds 4.7 eV. This enhancement can be attributed to the augmented disparity in energy levels between the back contact point material and the perovskite layer resulting from the increased work function of the back contact point. This improvement fosters better energy band alignment, thereby enhancing electron injection and transmission efficiency. In addition, the increase in the work function of the back contact point also helps to form a stronger charge selective contact, prevents the reflow of the charge, and further improves the efficiency of the battery. However, when the work function exceeds 4.7 eV, the performance of the battery tends to be stable. Even if the work function is increased, the device performance will not be significantly improved, which may be because the adjustment of the energy band structure is close to saturation. Hence, in the design of CsSnX_3_ all-inorganic perovskite devices, judiciously tuning the work function of the back contact point within a suitable range can significantly enhance the performance and efficiency of the cells. In the back electrode materials, the work functions of gold (Au), nickel (Ni), and carbon (C) are greater than or equal to 4.7 eV [19]. Among them, carbon (5.0 eV) as the back electrode material has the advantages of low cost, excellent electrical conductivity, and good chemical stability. It is an excellent material choice for the back electrode of CsSnX_3_ all-inorganic perovskite devices. However, carbon-based plating is difficult in the experiment, so it can be replaced by gold (Au). However, in order to find the best economy in the simulation prediction, carbon (5.0 eV) is selected as the back electrode of CsSnX_3_ all-inorganic perovskite devices.

### 2.3. The Effect of the Hole Transport Layer

Because the hole transport material has a certain selectivity, it can block the transmission of electrons and promote the transmission of holes, which can reduce the recombination phenomenon of the device. Therefore, hole materials play an important role in CsSnX_3_ (X = Cl, Br, I) all-inorganic perovskite devices [20]. When the photon is absorbed to produce a hole pair, the holes must be quickly transmitted to the back electrode of the battery to maintain the charge balance. Therefore, hole materials generally have high hole mobility and conductivity, which can quickly and effectively transport holes and reduce the composite of electron–hole pairs. Other than that, a good energy band alignment between the hole material and the absorbing layer is required to prevent electrons from transporting from the absorbing layer to the hole material. Therefore, in this study, organic hole materials (Spiro-MeOTAD, P3HT, CBTS) and inorganic hole materials (Cu_2_O, CuI, MoO_3_) were selected to explore the efficiency improvement of CsSnX_3_ all-inorganic perovskite devices.

**Figure 4:**
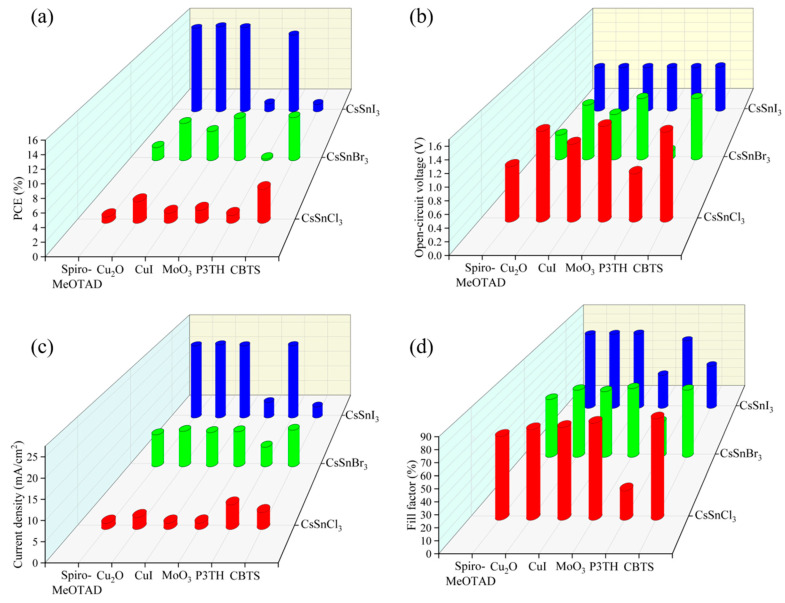
Different hole materials: (**a**) photoelectric conversion efficiency; (**b**) open-circuit voltage; (**c**) short circuit current; (**d**) fill factor.

By utilizing the physical parameters outlined in Table 3, the impact of various hole materials on the performance of CsSnX_3_ (X = Cl, Br, I) all-inorganic perovskite devices was investigated through the SCAPS-1D simulation software. From the simulation results of Figure 4a–d, it can be seen that when CBTS is used as a hole material, CsSnCl_3_ and CsSnBr_3_ all-inorganic perovskite solar cells show the best performance. For CsSnI_3_ all-inorganic perovskite devices, Cu_2_O is very suitable as its hole transport layer and achieves high photoelectric conversion efficiency. This discrepancy arises from variations in the energy band structure of CsSnX_3_ all-inorganic perovskite devices. Different hole materials have different energy bands matching with all-inorganic perovskite, thus affecting the performance of the device. Because CBTS and Cu_2_O have a good energy band matching relationship with CsSnX_3_ all-inorganic perovskite compared with other hole materials, the hole transport rate of the two materials is relatively large, which is conducive to hole transport. In addition, these two materials have a wide band gap, which can effectively block electrons and avoid the occurrence of the recombination phenomenon, thus enhancing the device’s performance and achieving the optimal PCE for CsSnX_3_ all-inorganic perovskite devices. In conclusion, the utilization of CBTS as a hole material yields optimal performance for CsSnCl_3_ and CsSnBr_3_ all-inorganic perovskite devices. Furthermore, Cu_2_O is the most effective hole transport material for all-inorganic perovskite solar cells incorporating CsSnI_3_. Consequently, this research will proceed with further optimization of CsSnX_3_ all-inorganic perovskite devices based on these findings.

### 2.4. The Effect of the Electron Transport Layer

In CsSnX_3_ all-inorganic perovskite devices, the electron transport material facilitates the transportation of electrons produced by the light-absorbing layer to the electrode. A high-quality electron transport layer enhances electron mobility and transmission efficiency while minimizing electron recombination losses [25]. In addition, the energy band structure of the electron transport layer and the alignment of energy bands in the light-absorbing layer (CsSnX_3_ all-inorganic perovskite) are also vital for electron transport and extraction. Appropriate band alignment helps to decrease the energy band barrier and improve the transmission efficiency of electrons, thereby promoting the performance of the device. Therefore, this study simulated the effects of organic electron transport materials (PCBM) and a range of inorganic electron transport materials (TiO_2_, ZnSe, CdS, STO, WS_2_) on the performance of CsSnX_3_ all-inorganic perovskite devices.

**Figure 5:**
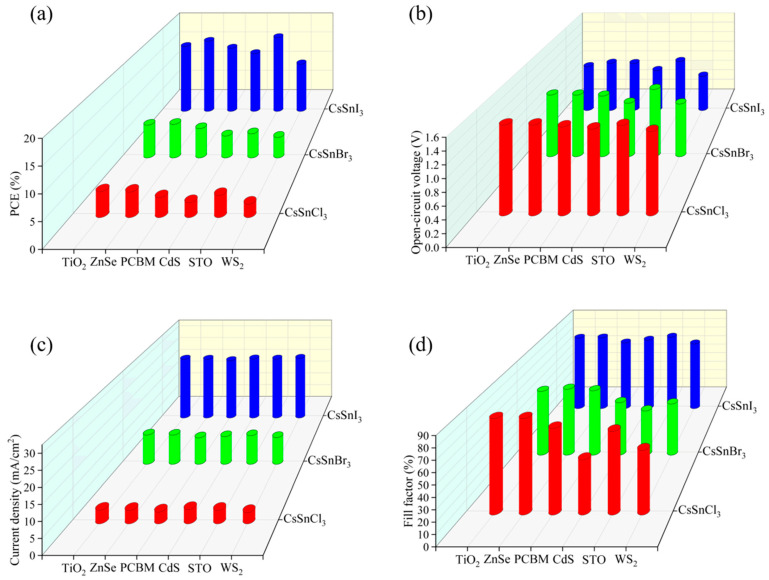
Different electronic materials: (**a**) photoelectric conversion efficiency; (**b**) open-circuit voltage; (**c**) short circuit current; (**d**) fill factor.

The effects of different electronic materials on the efficiency of CsSnX_3_ (X = Cl, Br, I) all-inorganic perovskite devices were simulated by using the physical parameters in Table 4. As depicted in Figure 5a–d, optimal performance was observed when TiO_2_ served as the electron layer material for CsSnCl_3_ all-inorganic perovskite devices. Relatively speaking, for CsSnBr_3_ all-inorganic perovskite devices, employing ZnSe as the electron layer material, facilitated excellent energy band alignment and high electron mobility, resulting in superior performance characteristics. For CsSnI_3_ all-inorganic perovskite devices, although the energy band matching between STO as an electron layer material and the CsSnI_3_ light absorption layer was not the best choice, the performance of CsSnI_3_ all-inorganic perovskite devices was greatly improved due to the high electron mobility of STO. In summary, this paper will use TiO_2_, ZnSe, and STO as the electron transport layers of CsSnCl_3_, CsSnBr_3_, and CsSnI_3_, respectively, to continue to optimize device performance.

### 2.5. The Effect of Defect Density

In the context of CsSnX_3_ all-inorganic perovskite devices, the density of defects is a critical factor that influences their operational efficiency [29]. Defect density mainly refers to the number of defects in the device structure, including structural defects, impurity defects, and surface defects. These defects will reduce the device’s performance [30].

**Figure 6:**
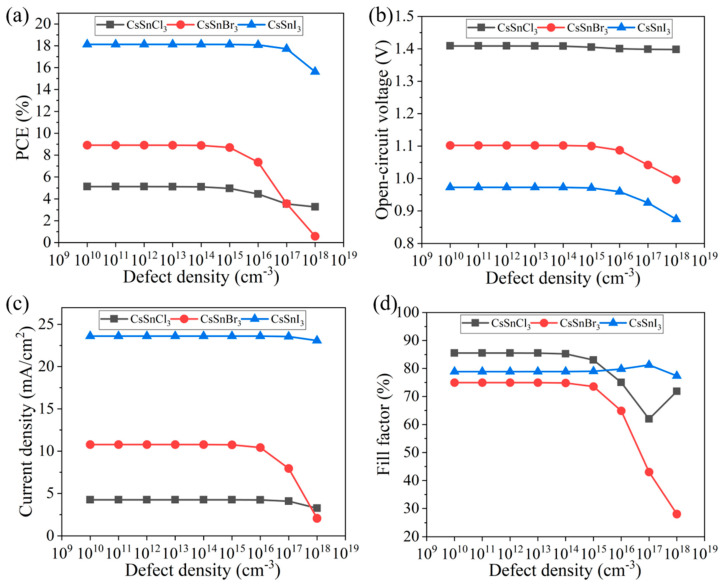
Defect density of different light absorption layers: (**a**) photoelectric conversion efficiency; (**b**) open-circuit voltage; (**c**) short circuit current; (**d**) fill factor.

When the defect density is too large, the performance of the CsSnX_3_ all-inorganic perovskite device will be significantly reduced. Therefore, reducing the defect density of the light absorption layer can improve the device’s efficiency. In this study, the effects of different defect concentrations (1 × 10^10^~1 × 10^18^ cm^−3^) on the performance of the CsSnX_3_ all-inorganic perovskite device were simulated, as shown in Figure 6a–d. According to the simulation results, when the defect density of the CsSnX_3_ (X = Cl, Br, I) optical absorption layer is ≤1 × 10^13^ cm^−3^, the efficiency change of the device tends to be stable and reaches the maximum, and the device performance starts to decline outside of this range. This is because the higher defect density will lead to energy level impurities or structural defects in the absorption layer, which may cause light scattering, reflection, or absorption loss and reduce the utilization efficiency of light energy. In addition, too high a defect density will also cause the recombination of electrons and holes, reducing the separation efficiency of photogenerated carriers and the transmission efficiency of electrons, thus reducing the overall performance of the device. Therefore, the defect density of CsSnX_3_ all-inorganic perovskite can be controlled below 1 × 10^13^ cm^−3^ during the experimental preparation process to ensure the best performance of the device.

### 2.6. The effect of the Light-Absorbing Layer’s Thickness

The thickness of the light-absorbing layer is an important factor in solar cells which directly affects the optical absorption and solar cell efficiency [31,32]. When the optical absorption layer is thin, the light can more easily penetrate the material, speed up the absorption of light, and thus improve the solar cell efficiency. In addition, a thinner light-absorbing layer can also reduce material costs and improve the manufacturing economy. However, when the light absorption layer is too thin, it may not be able to fully absorb the energy emitted, resulting in energy loss. On the contrary, when the light-absorbing layer is thick, the path length of light in the material increases, improving the absorption opportunity of light, making photons easier to capture, thus improving the solar cell efficiency of devices. However, an excessively thick light-absorbing layer can also increase scattering and reflection inside the material, causing light to propagate back and forth in the material and not be fully absorbed. In addition, an excessively thick light-absorbing layer may also lead to material waste. Therefore, when designing the light absorption layer, it is necessary to consider the relationship between the thickness of the optical absorption layer and the light absorption efficiency.

**Figure 7:**
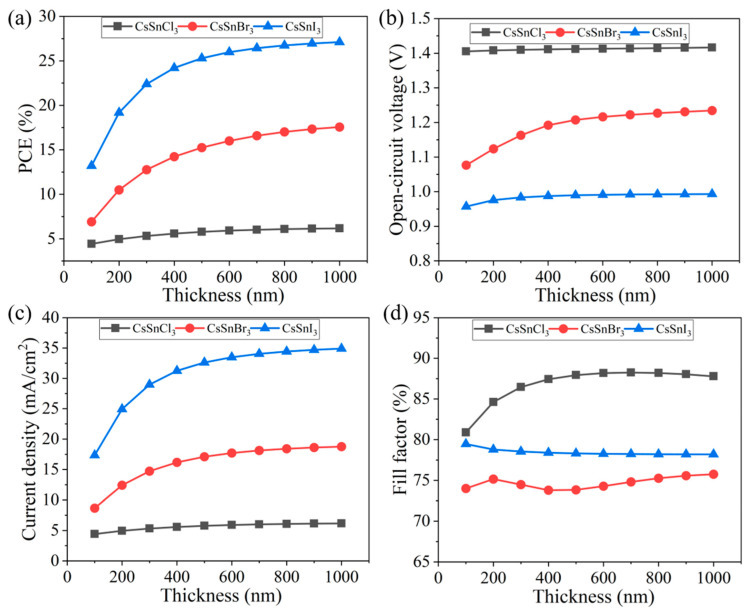
Different thicknesses of light absorption layer: (**a**) photoelectric conversion efficiency; (**b**) open-circuit voltage; (**c**) short circuit current; (**d**) fill factor.

In this study, the effects of different thicknesses (100~1000 nm) of the light absorption layer on the performance of the CsSnX_3_ all-inorganic perovskite device were explored, as shown in Figure 7a–d. The thickness mainly affects the current density of the device (CsSnCl_3_ increased by 1.07 mA/cm^2^, CsSnBr_3_ increased by 10.09 mA/cm^2^, CsSnI_3_ increased by 17.55 mA/cm^2^), resulting in a change in the PCE of the device. The PCE of the CsSnX_3_ all-inorganic perovskite device gradually increases with the thickness. This is because when the thickness is thinner, the photon is more easily absorbed, generating photo-generated carriers, thereby improving efficiency. Nevertheless, once the device thickness surpasses a certain threshold, the PCE of CsSnX_3_ all-inorganic perovskite device tends to stabilize. This phenomenon could be attributed to the likelihood that when the light absorption layer reaches a specific thickness, photon absorption may attain saturation. Consequently, further increases in device thickness may not yield significant efficiency enhancements.

The rationale behind selecting a thickness range up to 1000 nm stems from the balance between photon absorption and charge transport properties within the perovskite material. When the perovskite layer is thin, photon absorption is insufficient, leading to fewer photo-generated carriers and lower current density. As the thickness increases, more photons are absorbed, generating more carriers and increasing the current density and PCE. However, beyond a certain thickness, the mean free path of charge carriers becomes a limiting factor. The mean free path is the average distance a carrier travels before scattering, which is crucial for efficient charge collection.

In summary, by selecting the thickness of the light absorption layer within the appropriate thickness range, the effective absorption of light and the maximization of PCE can be achieved, while avoiding material waste and resource loss. In this study, the thickness of the CsSnX_3_ all-inorganic perovskite layer was set to 800 nm to optimize device performance.

### 2.7. The Effect of Temperature

**Figure 8:**
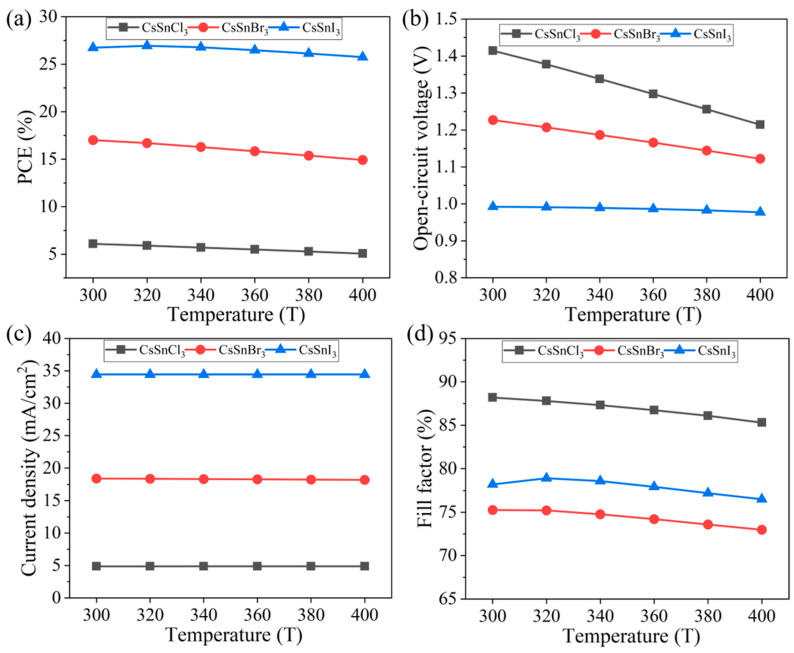
Different temperatures: (**a**) photoelectric conversion efficiency; (**b**) open-circuit voltage; (**c**) short circuit current; (**d**) fill factor.

In the advancement of all-inorganic perovskite technology, enhancing the thermal stability of materials stands out as a primary challenge, particularly for all-inorganic perovskite materials like CsSnX_3_. Hence, this study investigated the impact of temperature on the CsSnX_3_ all-inorganic perovskite device, simulating the variation in device performance under high-temperature conditions ranging from 300 K to 400 K, as depicted in Figure 8a–d.

According to the simulation results, with the increase in temperature, the performance of CsSnX_3_ all-inorganic perovskite device shows a linear downward trend, but the decrease is small (the electrical conversion efficiency of CsSnCl_3_ decreased by 0.12%, and the initial efficiency ratio still maintained about 83% efficiency; the electrical conversion efficiency of CsSnBr_3_ decreased by 2.09%, and the efficiency ratio to the initial efficiency remained about 87%. The electrical conversion efficiency of CsSnI_3_ decreased by 0.99%, and the efficiency ratio to the initial efficiency remained about 96%). This further proves that all-inorganic perovskite materials have higher thermal stability as light absorption layers than organic–inorganic hybrid materials. The decrease in open-circuit voltage with the increase in temperature can be explained by Formula (6), where E_g/q_ is always greater than V_OC_, indicating that the gradient of open-circuit voltage to temperature is negative, so the open-circuit voltage of CsSnX_3_ (X = Cl, Br, I) all-inorganic perovskite devices will decrease with the increase in temperature [33]. This is also the main reason why the performance of the device decreases with increasing temperature.

(1) $$\frac{d(V_{OC})}{dT}=\frac{V_{OC}}{T}-\frac{E_{g/q}}{T}$$

In the formula, $V_{OC}$—open-circuit voltage, V; T—temperature, K; $E_{g/q}$—the band gap energy of materials, eV.

## 3. Discussion

**Figure 9:**
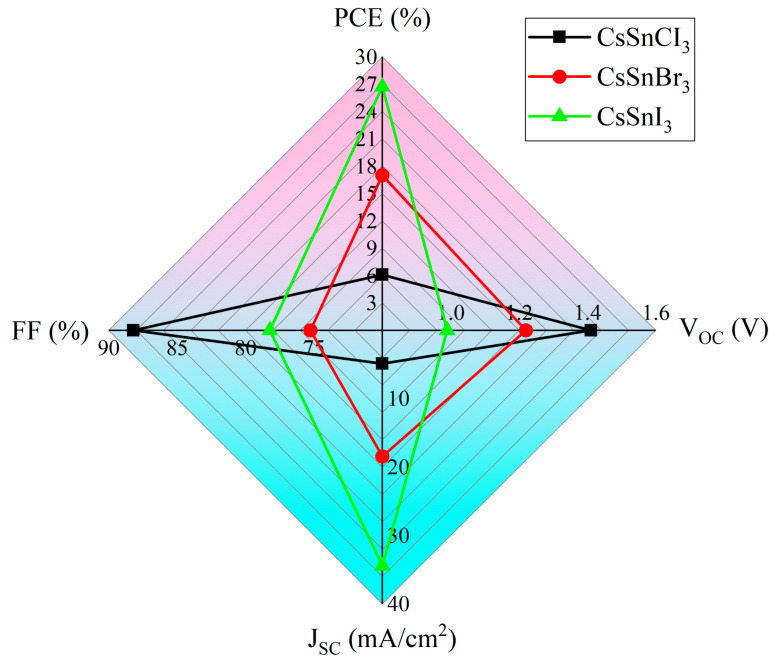
Performance comparison of different X-site element devices.

Following optimization, the performance of the CsSnX_3_ all-inorganic perovskite device was enhanced to different extents. By comparing the physical parameters in Table 5, it can be found that the optimized all-inorganic perovskite device improved its performance parameters. However, under the same conditions (such as defect density, optical absorption layer thickness, etc.), the change in different X-site elements has different effects on device performance. According to Table 5 and Figure 9, when the X-site elements are replaced by Cl, Br, and I in turn, the device’s performance increases in turn. This is because the band gap of CsSnX_3_ gradually narrows with the substitution of X-site elements from Cl to I. The change in the band gap width will affect the light absorption range of the material and the separation efficiency of the photogenerated carriers, thus affecting the photoelectric conversion efficiency. In general, materials with narrower band gaps have stronger absorption of visible light, which is conducive to the generation and transmission of photogenerated carriers and improves the PCE. In addition, the lattice constants of CsSnX_3_ (X = Cl, Br, I) may increase slightly with the substitution of X-site elements from Cl to I, resulting in an improvement in lattice matching. Good lattice matching helps to reduce the defect density and improve the carrier mobility and lifetime, thereby further improving the PCE, which leads to the performance of the device (efficiency: CsSnCl_3_ < CsSnBr_3_ < CsSnI_3_).

At present, the research simulation of CsSnX_3_ also achieves good development. Hossain’s group used SCAPS-1D simulation software to optimize the electron transport layer and hole transport layer of CsSnCl_3_ perovskite solar cells. Because different materials have different energy band matching with the perovskite layer, different material combinations will lead to different photoelectric conversion efficiencies of the device. Finally, they determined the best device structure is ITO/PCBM/CsSnCl_3_/CBTS and achieved the best performance [34]. The Seyed-Talebi group successfully achieved a device with a photoelectric conversion efficiency of 21.63% by controlling the thickness and temperature of CsSnI_3_ [35]. The hole transport of the device was further doped and optimized by SCAPS-1D, so the performance of CsSnI_3_ all-inorganic perovskite solar cells was significantly improved, reaching a photoelectric conversion efficiency of 23.63% [36]. Khatoon’s group also optimized CsSnBr_3_ all-inorganic perovskite solar cells based on SCAPS-1D and finally achieved a solar device with a photoelectric conversion efficiency of 17.06% [37].

In addition to the above, we compared the simulation results with the published experimental results, as shown in Table 6. Although the photoelectric conversion efficiency of Cl is 3.57% lower, the photoelectric conversion efficiency of Br and I is 6.56% and 13.78% higher than the published experimental results, respectively. These findings suggest that by optimizing the halogen elements in CsSnX_3_, device performance can be significantly improved. This not only provides valuable theoretical guidance for the development of highly efficient all-inorganic perovskite solar cells but also has important implications for practical applications, as this optimization helps to achieve higher photoelectric conversion efficiency and more stable device performance.

## 4. Materials and Methods

### 4.1. Introduction to Simulation Software

SCAPS-1D (Solar Cell Capacitor Simulator-1D) stands as a prominent simulation software tool extensively utilized in the solar cell domain [38]. SCAPS-1D uses a one-dimensional electron transport model to effectively simulate and refine the photoelectric characteristics of solar cells, taking into account key processes such as carrier drift, diffusion, and recombination, thus reflecting the characteristics of devices under different operating conditions. The advantages of this software include a simplified model, saving computing resources, and wide applicability, but it also has some disadvantages such as over-simplification, ignoring side effects, and limited application range. Although the one-dimensional model has certain limitations, it is an effective tool in many cases to simulate and analyze the motion and behavior of electrons in optoelectronic devices. In addition, SCAPS-1D also supports the setting of interface characteristics between different materials, such as interface defects and defect types, and it provides a series of optimization algorithms to calculate the current–voltage characteristics, band structure, and spectral response of solar cells by solving three interrelated differential equations: the Poisson equation, the continuity equation, and the drift–diffusion equation. The three coupled differential equations of SCAPS-1D are as follows [39,40]:

Poisson equation:

(2) $$\frac{\partial^{2}}{\partial x^{2}}\varphi (x)=\frac{q}{\varepsilon }[n(x)-p(x)-N_{D}^{+}(x)+N_{A}^{-}(x)-P_{t}(x)+N_{t}(x)]$$

The electron and hole continuity equations are as follows:

(3) $$\frac{\partial J_{n}}{\partial x}-U_{n}+G_{n}=0$$

(4) $$-\frac{\partial J_{p}}{\partial x}-U_{p}+G_{p}=0$$

For electrons and holes, the drift–diffusion equations are as follows:

(5) $$J_{n}=qn(x)\mu_{n}E(x)+qD_{n}\frac{dn}{dx}$$

(6) $$J_{p}=qn(x)\mu_{p}E(x)+qD_{p}\frac{dp}{dx}$$

The parameters in the formula are expressed as φ—potential (V), q—charge (C), ε—dielectric constant (F/m), n—electron density (cm^−3^), p—hole density (cm^−3^), $N_{D}^{+}$—donor doping density (cm^−3^), $N_{A}^{-}(x)$—acceptor doping density (cm^−3^), $P_{t}$—hole defect density (cm^−3^), $N_{t}$—electron defect density (cm^−3^), $J_{n}$—electron current density (cm^−3^), $J_{p}$—hole current density (cm^−3^), $U_{n}$—electron recombination rate (cm^3^/s), $U_{p}$—hole recombination rate (cm^3^/s), $G_{n}$—electron production rate (cm^3^/s), $G_{p}$—hole generation rate (cm^3^/s), $\mu_{n}$—electron mobility (cm^2^·V^−1^·s^−1^), $\mu_{p}$—hole mobility (cm^2^·V^−1^·s^−1^), E(x)—electric field strength (V/cm), $D_{n}$—electron diffusivity (cm^2^/s), and $D_{p}$—hole diffusion rate (cm^2^/s).

**Figure 10:**
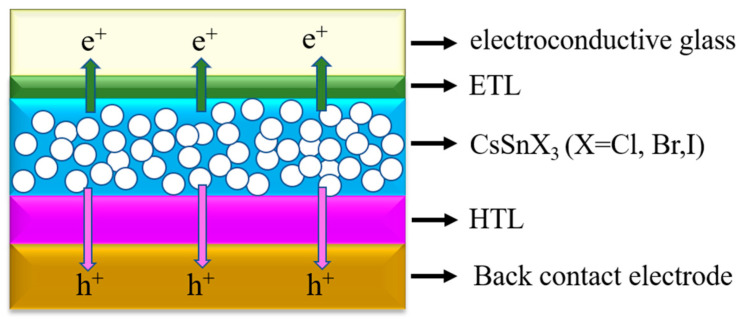
CsSnX_3_ (X = Cl, Br, I) device configuration structure in simulation.

Based on an accurate electron transport model, SCAPS-1D can accurately simulate the performance of CsSnX_3_ (X = Cl, Br, I) all-inorganic perovskite devices [34]. It considers the key factors such as the energy band structure, carrier migration, and recombination of the material, and it can accurately predict the efficiency of the battery. SCAPS-1D is equipped with an efficient optimization algorithm, which can search for the best combination of parameters in a short time. By iterating multiple times, the efficiency of CsSnX_3_ all-inorganic perovskite devices can be enhanced gradually, leading to the discovery of the optimal device design. In addition, SCAPS-1D allows users to customize the parameters of all-inorganic perovskite materials, such as band width, carrier mobility, absorption coefficient, and surface reflectivity. This allows researchers to simulate and optimize the performance of CsSnX_3_ all-inorganic perovskite devices based on specific experimental material data or theoretical predictions. SCAPS-1D not only generates current–voltage (J-V) characteristic curves for solar cells but also furnishes comprehensive result analysis, aiding researchers in comprehending the operational principles of CsSnX_3_ all-inorganic perovskite devices. (In Figure 10, the simulation adopts the forward plane structure device.)

### 4.2. Introduction of Physical Parameters

Physical parameters have an important impact on the performance of all-inorganic perovskite devices [41,42]. The band gap (E_g_) is a key parameter that determines the absorption range of the solar spectrum. A wide band gap can absorb high-energy photons, but it will reduce the absorption range. A narrower band gap can absorb more sunlight but is susceptible to the loss of hot electrons. Therefore, when designing all-inorganic perovskite devices, the choice of band gap is crucial to ensure excellent photoelectric conversion efficiency. In addition to the band gap, the interface properties and other physical parameters listed in Table 1 are also critical to the optimization of CsSnX_3_ (X = Cl, Br, I) all-inorganic perovskite devices. These parameters interact with each other and together affect device performance, so they need to be considered in the design and optimization process.

## 5. Conclusions

In this study, SCAPS-1D numerical simulation software was used to systematically optimize and numerically simulate all-inorganic perovskite devices based on the CsSnX_3_ (X = Cl, Br, I) light absorption layer. The optimal performance of the CsSnX_3_ all-inorganic perovskite device was successfully simulated under the same conditions by optimizing the device’s work function, hole transport material, electron transport material, defect density, and light absorption layer thickness.

When the hole and electron materials of CsSnCl_3_ are CBTS and TiO_2_, respectively, the hole and electron materials of CsSnBr_3_ are CBTS and ZnSe, respectively, and the hole and electron materials of CsSnI_3_ are Cu_2_O and STO, respectively, the device can obtain higher performance, which is due to their good energy band matching relationships. In addition, when the FTO work function of the conductive glass is ≤4.54 eV, the work function of the back electrode is ≥4.54 eV, and the defect density and thickness of the light absorption layer are ≤1 × 10^13^ cm^−3^ and ≥800 nm, respectively, the performance of CsSnX_3_ all-inorganic perovskite device is optimal. With the successive substitution of halogen elements (Cl, Br, I), the theoretical PCE of the device reached 6.09%, 17.02%, and 26.74%, respectively. Its performance showed a significant increasing trend with the single substitution of X-site elements. Among them, the CsSnI_3_ all-inorganic perovskite device achieved a photoelectric conversion efficiency of 26.74%, mainly due to its small band gap and wide light absorption range. By adjusting the thickness of the CsSnI_3_ perovskite layer (100~1000 nm), the current density can be effectively increased, thereby improving the PCE of the device. Based on these theoretical results, it provides researchers with good theoretical guidance and is expected to achieve optimal device performance in the laboratory by adjusting process parameters.

## Figures and Tables

**Table 3:** Physical parameters of hole transport layer.

Parameters	Spiro-MeOTAD	Cu_2_O	CuI	MoO_3_	P3TH	CBTS
Thickness (nm)	100	100	100	100	100	100
Energy band E_g_/(eV)	2.8	2.17	3.1	3.0	1.8	1.9
Electron affinity χ/(eV)	2.05	3.2	2.1	2.5	3.1	3.6
Relative permittivity ε_r_	3	7.11	6.5	12.5	3.4	5.4
Effective conduction band density N_c_/(cm^−3^)	2.2 × 10^18^	2.02 × 10^17^	2.8 × 10^19^	2.2 × 10^18^	1 × 10^22^	2.2 × 10^18^
Effective valence band density N_v_/(cm^−3^)	1.8 × 10^19^	1.1 × 10^19^	1 × 10^19^	1.8 × 10^19^	1 × 10^22^	1.8 × 10^19^
Electron mobility μ_n_/(cm^2^·V^−1^·s^−1^)	0.0002	200	100	25	0.0001	30
Hole mobility μ_p_/(cm^2^·V^−1^·s^−1^)	0.0002	80	43.9	10	0.001	10
Donor density N_D_/(cm^−3^)	0	0	0	0	0	0
Receptor density N_A_/(cm^−3^)	1.0 × 10^18^	1 × 10^18^	1 × 10^18^	1 × 10^18^	3.17 × 10^13^	1 × 10^18^
Defect density N_t_/(cm^−3^)	1.0 × 10^15^	1 × 10^15^	1 × 10^15^	1 × 10^15^	1 × 10^14^	1 × 10^15^
Reference	[15]	[21]	[21]	[22]	[23]	[24]

**Table 4:** Physical parameters of electron transport layer.

Parameters	TiO_2_	ZnSe	PCBM	CdS	STO	WS_2_
Thickness (nm)	25	25	25	25	25	25
Energy band E_g_/(eV)	3.2	2.81	2	2.4	3.2	1.87
Electron affinity χ/(eV)	4.0	4.09	3.9	4.18	4	4.3
Relative permittivity ε_r_	9.0	8.6	3.9	10	8.7	11.9
Effective conduction band density N_c_/(cm^−3^)	1.0 × 10^21^	2.2 × 10^18^	2.5 × 10^21^	1.9 × 10^19^	1.7 × 10^19^	2.4 × 10^19^
Effective valence Band density N_v_/(cm^−3^)	2.0 × 10^20^	1.8 × 10^18^	2.6 × 10^21^	2.2 × 10^18^	2 × 10^20^	1 × 10^19^
Electron mobility μ_n_/(cm^2^·V^−1^·s^−1^)	20	400	0.2	100	5300	260
Hole mobility μ_p_/(cm^2^·V^−1^·s^−1^)	10	110	0.2	10	660	51
Donor density N_D_/(cm^−3^)	1 × 10^18^	1 × 10^18^	2.93 × 10^17^	1 × 10^17^	2 × 10^16^	1 × 10^16^
Receptor density N_A_/(cm^−3^)	0	0	0	0	0	0
Defect density N_t_/(cm^−3^)	1 × 10^15^	1 × 10^15^	1 × 10^15^	1 × 10^15^	1 × 10^15^	2 × 10^15^
Reference	[12]	[26]	[23]	[26]	[27]	[28]

**Table 5:** Optimized physical characteristic parameters.

Device Structure	V_OC_ (V)	J_SC_ (mA/cm^2^)	FF (%)	PCE (%)
FTO(4.54)/TiO_2_/CsSnCl_3_/CBTS/C(5.0)	1.41	4.88	88.19	6.09
FTO(4.54)/ZnSe/CsSnBr_3_/CBTS/C(5.0)	1.22	18.43	75.25	17.02
FTO(4.54)/STO/CsSnI_3_/Cu_2_O/C(5.0)	0.99	34.44	78.22	26.74

**Table 6:** Simulation results are compared with experimental results.

Theoretical and Experimental	Device	PCE (%)	References
This article’s simulation	CsSnCl_3_	6.09	
	CsSnBr_3_	17.02	
	CsSnI_3_	26.74	
Experiment	CsSnCl_3_	9.66	[10]
	CsSnBr_3_	10.46	
	CsSnI_3_	12.96	

## Data Availability

Data are contained within the article.

## Abbreviations

Spiro-OMeTAD	2,2′,7,7′-tetrakis[N,N-bis(4-methoxyphenyl)amino]-9,9′-spirobis Fluorene
P3HT	Poly(3-hexylthiophene-2,5-diyl)
CBTS	C_60_Br_24_S_66_
Cu_2_O	cuprous oxide
CuI	cuprous iodide
MoO_3_	molybdenum trioxide
PCBM	[6,6]-phenyl-C61-Isomethylbutyrate
TIO_2_	titanium dioxide
ZnSe	tin oxide
CdS	Cadmium sulfide
STO	Strontium titanate
WS_2_	Tungsten disulfide
PCE	Photoelectric conversion efficiency
V_OC_	Pen circuit voltage
J_SC_	Short circuit current
FF	Fill factor
